# Supplementary material for: The Systems Biology Research Tool: evolvable open-source software
Source: BMC Syst Biol. 2008 Jun 29;2:55. doi: 10.1186/1752-0509-2-55 (PMC2446383; doi:10.1186/1752-0509-2-55)
Supplement: Additional file 1 — SBRT Archive. An archive of the current version of the Systems Biology Research Tool. [file 1752-0509-2-55-S1.zip › sbrt-1.4.0/doc/developers_guide/api/sbrt/kernel/math/geom/class-use/HashConvexPolytope.html]

Uses of Class sbrt.kernel.math.geom.HashConvexPolytope


|  |  |  |
| --- | --- | --- |
| **Overview** \| **Package** \| **Class** \| **Use** \| **Tree** \| **Deprecated** \| **Index** \| **Help** | |  |
| PREV   NEXT | **FRAMES**    **NO FRAMES**     **All Classes** |  |


---


## **Uses of Class sbrt.kernel.math.geom.HashConvexPolytope**

| Packages that use HashConvexPolytope | |
| --- | --- |
| **sbrt.kernel.math.geom** | Provides classes and interfaces for creating objects relevant to computational geometry. |

Uses of HashConvexPolytope in sbrt.kernel.math.geom

Methods in sbrt.kernel.math.geom that return HashConvexPolytope

`static

<V> HashConvexPolytope<V>`

`HashConvexPolytope.make(LinearSystemSolution<V> systemSolution,
java.util.Map<? extends V,Interval> variableBounds,
double tolerance)`
  
          Constructs a new convex poltyope from the provided linear system solution
and variable bounds.

---


|  |  |  |
| --- | --- | --- |
| **Overview** \| **Package** \| **Class** \| **Use** \| **Tree** \| **Deprecated** \| **Index** \| **Help** | |  |
| PREV   NEXT | **FRAMES**    **NO FRAMES**     **All Classes** |  |


---
